# The Changes of Functional Connectivity Strength in Electroconvulsive Therapy for Depression: A Longitudinal Study

**DOI:** 10.3389/fnins.2018.00661

**Published:** 2018-09-25

**Authors:** Qiang Wei, Tongjian Bai, Yang Chen, Gongjun Ji, Xiaopeng Hu, Wen Xie, Zulun Xiong, Daomin Zhu, Lin Wei, Panpan Hu, Yongqiang Yu, Kai Wang, Yanghua Tian

**Affiliations:** ^1^Department of Neurology, The First Affiliated Hospital of Anhui Medical University, Hefei, China; ^2^Collaborative Innovation Centre of Neuropsychiatric Disorders and Mental Health, Hefei, China; ^3^Anhui Mental Health Center, Hefei, China; ^4^Department of Radiology, The First Affiliated Hospital of Anhui Medical University, Hefei, China

**Keywords:** depression, electroconvulsive therapy, fMRI, treatment, brain hub

## Abstract

Electroconvulsive therapy (ECT) is an effective treatment for depression, but the mechanism of ECT for depression is still unclear. Recently, neuroimaging studies have reported that the prefrontal cortex, hippocampus, angular gyrus, insular and other brain regions are involved in the mechanism of ECT for depression, and these regions are highly overlapped with the location of brain hubs. Here, we try to explore the effects of ECT on the functional connectivity of brain hubs in depression patients. In current study, depression patients were assessed at three time points: prior to ECT, at the completion of ECT and about 1 month after the completion of ECT. At each time point, resting-state functional magnetic resonance imaging, assessment of clinical symptoms and cognition function were performed respectively, which was compared with 20 normal controls. Functional connectivity strength (FCS) was used to identify brain hubs. The results showed that FCS of left angular gyrus in depression patients significantly increased after ECT, accompanied by improved mood. The changed FCS in depression patients recovered obviously at 1 month after the completion of ECT. It suggested that ECT could modulate functional connectivity of left angular gyrus in depression patients.

## Introduction

Depression is a major cause of disability and global disease burden worldwide, contributing significantly to decrease in quality of life and increase in suicide risk (Ustun et al., 2004). Even with adequate psychotherapeutic, psychopharmacologic or combined treatment, about one third of patients do not achieve symptom remission (Rush et al., 2006). For these severely depressed and treatment-resistant patients, electroconvulsive therapy (ECT) elicits a more rapid and effective response, leading to remission of symptoms in about 50–70% of patients (Husain et al., 2004). However, the mechanism by which ECT operates is still unclear. Exploring the therapeutic mechanism of ECT would promote the optimization of ECT and develop new treatment options for depression.

Recently, neuroimaging studies have reported that the prefrontal cortex, hippocampus, angular gyrus, insular, and other brain regions are involved in the mechanism of ECT for depression (Perrin et al., 2012; Abbott et al., 2014; van Waarde et al., 2015; Cano et al., 2016; Sartorius et al., 2016). These results have showed that ECT does not only affect one brain region, but also change the structure and function of wide brain areas, which indicates that ECT may influence the brain at the level of brain networks. For brain networks, studies reveal topological organization of human whole-brain connectivity by combining neuroimaging and graph-based network analysis (Bullmore and Sporns, 2009; He and Evans, 2010). One important and convergent finding is that there are several hubs in the brain, located in prefrontal cortex, hippocampal, inferior parietal lobule (angular gyrus and supramarginal gyrus), and insular. These brain hubs have a significantly larger number of structural and functional connections in comparison with other regions in the brain network, and play important roles in information communication and integration across a broad range of cognitive tasks, such as emotion and cognitive control (van den Heuvel and Sporns, 2013; Mears and Pollard, 2016). The locations of hubs are highly overlapped with changed regions observed in previous studies about ECT for depression, and it could be hypothesized that the changed organization of brain hubs may be crucial for the effects of ECT for depression. But it is still lack of studies to test the hypothesis.

Here, we try to explore the effects of ECT on the functional connectivity of brain hubs in depression patients by using resting-state functional magnetic resonance imaging (rsfMRI) and functional connectivity strength (FCS). RsfMRI measures spontaneous brain activity. It has emerged as an effective probe for functional connectivity in both healthy individuals and neuropsychiatric patients. FCS is a voxel-wise measurement of ‘degree centrality’ for the whole-brain functional connectivity based on the rsfMRI data, which could reflect the general functional connections of brain hubs. FCS has been used to observe the organization of brain hubs in neuropsychiatric patients, including depression (Lan et al., 2015; Wang et al., 2015; Zou et al., 2016).

In the present study, depression patients were assessed at three time points: prior to ECT, at completion of ECT and about 1 month after the completion of ECT. At the three time points, rsfMRI, the assessment of clinical symptoms, and measures of cognitive function were performed.

## Materials and Methods

### Participants

Depression patients who were referred for ECT due to resistance to drug therapy or severe suicidal tendencies were recruited from Anhui Mental Health Center from September 2012 to December 2016. Depression was diagnosed on the basis of Diagnostic and Statistical Manual of Mental Disorders-IV criteria. Patients with substance dependence, pregnancy, life-threatening somatic disease, neurological disorders, other co-morbid mental disorders, MRI-related contraindications or head translation or rotation parameters exceeding 3 mm or 3° during MRI scanning were excluded. 26 right-handed patients were included in the final sample. All of them continued to take anti-depression drugs during ECT administration. At the same time, 9 patients also took antipsychotic medications and 3 patients took lithium. During the 1-month follow-up, patients continued to take anti-depression drugs. Patients were required to stop taking benzodiazepines 12–24 h before the first assessment point prior to ECT in the study.

Twenty normal controls were recruited who met the same exclusion criteria as the depression patients, except for a history of depression or antidepressant use. All the participants were right-handed.

The study was approved by the Anhui Medical University Ethics Committee, and all the participants in this study were undertaken with the understanding and written consents.

### Procedures

Patients were assessed at 3 time points: TP1, 12–24 h before the first ECT administration; TP2, 24 h-1 week after the last ECT administration; and TP3, about 1 month after the last ECT administration. All the 26 patients completed TP1 and TP2 assessments, and 15 patients completed the T3 assessments. All patients maintained the drug treatments during the study. Healthy controls completed one assessment. The assessments included a clinical evaluation, cognitive tests and magnetic resonance imaging (MRI) scan.

### ECT Administration

All the ECT administrations were conducted at Anhui Mental Health Center, where patients underwent modified bifrontal ECT with a Thymatron System IV Integrated ECT Instrument (Somatics, Inc., Lake Bluff, IL, United States). The first three ECT sessions occurred on consecutive days, and the remaining ECT sessions were conducted every other day with a break of weekends until patients achieved remission. The patient’s age determined the stimulating intensity of ECT. If the patient was younger than 50, the initial percent energy dial was set as patient’s age minus five (e.g., 43% for a 48 year-old patient); and if not, the initial percent energy dial setting was to the patient’s age (e.g., 55% for a 55 year-old patient). Seizure activity was monitored with electroencephalography during ECT. If no seizure activity resulted from stimulation, the initial percent energy would be increased until a therapeutically satisfactory seizure activity was obtained. During each ECT administration, patients were anesthetized with propofol. Succinylcholine and atropine were used to relax muscles and suppress gland secretions.

### Clinical Evaluation and Cognitive Tests

We used the 17-item Hamilton Depression Rating Scale (HAMD) to assess depressive symptoms for depression patients and health controls. Mini mental state examination (MMSE) was used to assess participants’ general cognitive function. A verbal fluency test was used to assess executive functions with patients asked to say as many words as possible within 2 min (1 minutes for animals, 1 min for vegetables). The total number of words was recorded, excluding repetitions, and intrusions.

### MRI Data Acquisition

All the participants in this study underwent the MRI scans at the First Affiliated Hospital of Anhui Medical University. All the participants were instructed to keep their eyes closed and to relax, to remain awake, and not to think of anything in particular during the scanning. All scans were performed with a clinical 3-T whole-body MRI scanner (Signa HDxt 3.0T, GE Healthcare, Little Chalfont, Buckinghamshire, United Kingdom) and used a standard echo planar imaging sequence. T1-weighted anatomic images were acquired in sagittal orientation by using a 3D inversion recovery prepared fast spoiled gradient recalled sequence (TR/TE = 8.676 ms/3.184 ms, inversion time = 800 ms, flip angle = 8 degrees, field of view = 256 × 256 mm^2^, matrix size = 256 × 256, slice thickness = 1 mm, voxel size = 1 × 1 × 1 mm^3^, sections = 188). The rsfMRI were recorded aligned along the anterior–posterior commissure line with the following parameters: repetition time/echo time ratio (TR/TE) = 2000 ms/22.5 ms, flip angle = 30 degrees, 33 slices, thickness/gap ratio = 4.0 mm/0.6 mm, voxel size = 3.4 × 3.4 × 4.6 mm^3^, matrix size = 64 × 64, field of view = 220 × 220 mm^2^.

### Data Processing

#### Functional Image Preprocessing

Data Processing Assistant for Resting-State Functional MR Imaging toolkit (DPARSF) was used to preprocess functional images, which synthesizes procedures in Resting State Functional MR Imaging Toolkit (REST^1^) and statistical parametric mapping software package (SPM8 ^2^) (Chao-Gan and Yu-Feng, 2010; Song et al., 2011). The first 10 volumes of data were discarded to ensure stable longitudinal magnetization. The remaining volumes were corrected for slice timing and head motion. All the participants had no more than 3 mm maximum displacement in any direction of x, y, or z and 3 degrees of angular motion. The individual T1 images were co-registered to functional images and were segmented (gray matter, white matter, and cerebrospinal fluid). After that, the functional images were normalized to the standard Montreal Neurological Institute space by using T1 image unified segmentation with a 12-parameter non-linear transformation, and resampled at a resolution of 3 × 3 × 3 mm^3^. Afterwards, functional images were linearly detrended and band-pass filtered (0.01–0.08 Hz) to reduce low-frequency drifts and physiological high-frequency noises. Finally, several sources of spurious covariance were linearly regressed, including the six motion parameters, and signals from white matter and cerebrospinal fluid.

#### Whole-Brain FCS Analysis

A voxel-wise whole-brain functional connectivity analysis was performed on the on the preprocessed rsfMRI data as follows. Firstly, the Pearson’s correlations between the residual time series of all pairs of brain voxels were computed and a whole-brain connectivity matrix for each participant was constructed. Then, the individual correlation matrices were transformed to a z-score matrix using a Fisher *r*-to-*z* transformation. For a given voxel, FCS was computed as the sum of the z-values between the given voxel and all the other voxels. We restricted our analysis to correlations above a threshold of *r* = 0.2 to eliminate weak correlations possibly arising from noises. These FCS maps were smoothed with a 6 mm full-width at half-maximum (FWHM) Gaussian kernel (Buckner et al., 2009; Liang et al., 2013).

### Statistical Analysis

Clinical and demographic data were analyzed by using SPSS 19.0 (SPSS, Inc., Chicago, IL, United States). Paired two-sample *t*-test was performed to determine the changed regions of FCS for depression patients between the first two time points-prior to ECT and at completion of ECT. This test was constrained in a gray matter (GM) mask, and multiple comparison corrections were based on Gaussian random field theory (voxel-level *p* < 0.001; cluster-level *p* < 0.05). The FCS data of the changed regions were extracted in normal controls and patients at all three time points-prior to ECT, at completion of ECT and about 1 month after the completion of ECT, to observe the dynamic change. Independent *t*-test was used for between group, and paired *t*-test was used for within-group comparisons. The comparisons between TP2 and TP3 were constrained to the patients who completed the procedure at TP3. Pearson correlation analyses were performed to assess the correlation of the changed behavior scores and changed values of FCS in patients between the three time points (the significance level: *p* < 0.05).

## Results

### Baseline Characteristics

The 26 patients with depression at TP1 and TP2 are matched with health controls in gender (*χ*2 = 0.033, *p* = 0.855), age (*t* = −0.796, *p* = 0.430), and education years (*t* = −0.555, *p* = 0.582). The 15 patients at TP3 and control groups also did not differ in gender (*p* = 0.738; fisher exact probability), age (*t* = −0.741, *p* = 0.464), and education years (*t* = −0.349, *p* = 0.730).

### Clinical and Cognitive Outcome

#### Depressive Symptom

**FIGURE 1:**
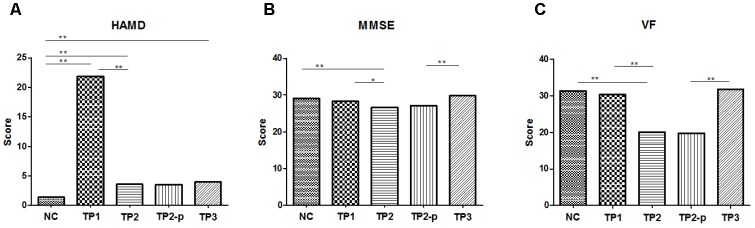
**(A)** The scores of HAMD in each group. **(B)** The scores of MMSE in each group. **(C)** The scores of VF in each group. The clinical and cognitive outcome in normal controls and depression patients. NC, normal controls; TP1, patients at 12–24 h before the first ECT administration; TP2, patients at 24 h-1 week after the last ECT administration; TP2_p, patients at TP2 who completed the sacn of TP3; TP3, patients at about 1 month after the last ECT administration; HAMD, 17-item Hamilton Depression Rating Scale; MMSE, Mini mental state examination; VF, Verbal fluency. ^∗^*p* < 0.05; ^∗∗^*p* < 0.01.

Compared to health controls (HC), patients at TP1 had significantly higher HAMD scores (*t* = 18.96, *p* < 0.001). Patients at TP2 show a significant reduction of mean HAMD score compared with TP1 (*t* = 18.84, *p* < 0.001), suggesting excellent treatment effect of ECT on depressive symptoms. No significant difference of HAMD score was observed between patients at TP2 and TP3 (*t* = 0.613, *p* = 0.550), which showed that the treatment effect of ECT on depressive symptoms was maintained. Both the HAMD scores of patients at TP2 and TP3, however, were still higher than health controls (TP2 vs. HC: *t* = 3.870, *p* < 0.001; TP3 vs. HC: *t* = 4.192, *p* < 0.001). (See in **Figure 1A**).

#### General Cognition

Compared to normal controls, MMSE scores were significantly lower in patients at TP2 (*t* = 3.788, *p* < 0.001), but no significant difference was observed in patients at TP1 (IR: *t* = 1.424, *p* = 0.162) and in patients at TP3 (*t* = 1.735, *p* = 0.092). MMSE scores showed a significant decrease in patients at TP2 compared with at TP1(*t* = 2.476, *p* = 0.017). At the follow-up assessments, there were significant improvements in MMSE scores at TP3 compared with at TP2 (*t* = 5.074, *p* < 0.001). (See in **Figure 1B**).

#### Executive Function

Compared to healthy controls, the VF score was significantly lower in patients at TP2 (*t* = 5.153, *p* < 0.001), but no significant difference was observed in patients at TP1 (*t* = 0.435, *p* = 0.665) and in patients at TP3 (*t* = 0.180, *p* = 0.859). The VF score showed a significant decrease in patients at TP2 compared with TP1 (*t* = 4.087, *p* < 0.001). This reduction was reversed at TP3 compared with at TP2 (*t* = 8.465, *p* < 0.001). (See in **Figure 1C**).

### ECT Effects on FCS in Depression Patients

**FIGURE 2:**
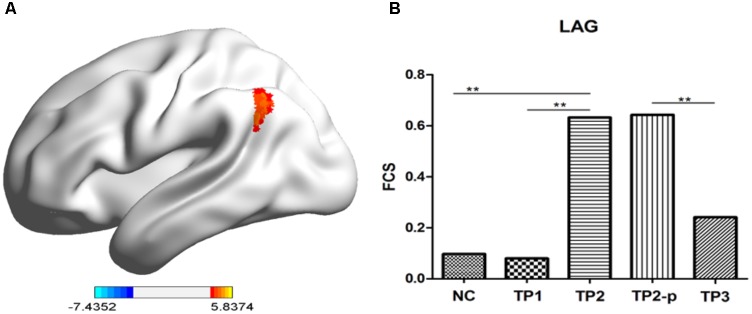
**(A)** The stereogram of LAG. **(B)** The FCS value of LAG in each group. The Functional connectivity strength (FCS) values of left angular gyrus in normal controls and depression patients. NC, normal controls; TP1, patients at 12–24 h before the first ECT administration; TP2, patients at 24 h-1 week after the last ECT administration; TP2_p, patients at TP2 who completed the sacn of TP3; TP3, patients at about 1 month after the last ECT administration; LAG, left angular gyrus. ^∗^*p* < 0.05; ^∗∗^*p* < 0.01.

To explore the effects of ECT on FCS, paired two-sample *t*-test was performed between the FCS maps between depression patients at TP1 and TP2 within the gray matter mask, correcting with Gaussian random field theory (voxel-level *p* < 0.001; cluster-level *p* < 0.05). The result showed that FCS was significantly increased in left angular gyrus in patients at TP2, compared to patients at TP1. (See in **Figure 2A** and **Table 1**).

**Table 1:** Regions showing significant changes in Functional connectivity strength (FCS) between depression patients at TP1 and TP2.

Brain regions	BA	Voxel number	*t* score	MNI coordinates (*x*, *y*, *z*)
Left angular gyrus	39	88	5.2981	−39, −60, 33

*BA, Brodmann area; One voxel, 3 × 3 × 3 mm3; t, statistical value of the peak voxel; MNI coordinates, Montreal Neurological Institute coordinates of the peak voxel.*

Then, to observe the dynamic changes of FCS data, the FCS values of the above cluster were extracted in health controls and patients at TP1, TP2, and TP3. Compared to healthy controls, no significant difference of FCS value was observed in left angular gyrus in patients at TP1 (*t* = 0.158, *p* = 0.875) and in patients at TP3 (*t* = 0.983, *p* = 0.333), but FCS of left angular gyrus in patients at TP2 was significantly higher than in healthy controls (*t* = 4.001, *p* < 0.001). FCS of of left angular gyrus wase significantly decreased in patients at TP3 compared with patients at TP2 (*t* = 3.046, *p* = 0.009). (See in **Figure 2B**).

### The Correlation Between the Changes of Depressive Symptom and FCS

No significant correlation was observed between the changes in the HAMD score and FCS value of the left angular gyrus for patients at TP1 and TP2 (*r* = 0.163, *p* = 0.427), as well as patients at TP2 and TP3 (*r* = 0.255, *p* = 0.358).

### The Correlation Between the Changes of Cognitive Outcomes and FCS

There was no significant correlation between the changes in cognitive outcomes (MMSE or VF) and FCS value of left angular gyrus for patients at TP1 and TP2 (MMSE: *r* = −0.105, *p* = 0.609; VF: *r* = −0.134, *p* = 0.515), as well as patients at TP2 and TP3 (MMSE: *r* = −0.274, *p* = 0.323; VF: *r* = −0.009, *p* = 0.975).

## Discussion

In this study, we observed changes of FCS in depression patients receiving ECT using a longitudinal protocol, and our main findings are 2-fold. First, ECT significantly increased the FCS of left angular gyrus in depression patients, accompanied by improved mood and impaired cognitive function. Second, the changed FCS in depression patients recovered obviously at 1 month after the completion of ECT, as well as the impaired cognitive function, but the improved mood did not worsen again. It has been demonstrated that left angular gyrus is a brain hub which has larger number of structural and functional connections with other brain regions (van den Heuvel and Sporns, 2013; Mears and Pollard, 2016), and FCS reflects general functional connections of brain hubs which has been temporarily changed after ECT in depression patients. These results have suggested that ECT could modulate the function of the brain hub (left angular gyrus) in depression patients.

Generalized epileptic seizure during ECT may contribute to the increased FCS in depression patients after ECT. When ECT is conducted, two electrodes are placed on patients’ skull, and electricity of 100–500 mC through electrodes were administrated to patients. The electricity would induce generalized epileptic seizure lasting tens of seconds (Lisanby, 2007). During generalized epileptic seizure, the neurons in the brain are activated simultaneously by the spread of electricity. So generalized epileptic seizure is abnormal synchronous neuronal activity in the brain (Grasse et al., 2013). In our study, FCS is an index of general functional connectivity, and it is fundamentally the correlation of Blood-oxygen-level dependent (BOLD) signals, and reflects the synchronization of neuronal activity among different brain regions. Generalized epileptic seizure may increase functional connectivity in the brain due to the synchronous neuronal activity, especially in the brain hubs, which has more structure and functional connections with other brain regions. So ECT could increase functional connectivity among brain hubs and other brain regions and caused increased FCS of left angular gyrus in depression patients. After the completion of ECT, synchronous neuronal activity during generalized epileptic seizure is not induced in depression patients, which would lead to gradually decrease of FCS in left angular gyrus.

Many studies have suggested that abnormality of left angular gyrus is closely related to depression. One study has reported that the volume of left angular gyrus is associated with suicide attempts in depression patients (Lee et al., 2016). It has been found that there is abnormal functional connectivity between left angular gyrus and precentral gyrus in depression patients (Wu et al., 2016). Recently, a whole brain functional connectome study has suggested that the FCS of left angular gyrus is lower in depression patients than in healthy controls (Lai et al., 2017). As a brain hub, angular gyrus process information by connecting distinct, functional specialized regions. These regions include amygdala, ventromedial prefrontal cortex, subgenual anterior cingulate cortex, and other areas which are related to emotion regulation (Achard et al., 2006). The lower FCS of left angular gyrus means less connections with other areas, which may influence emotion regulation and cause depression. In current study, the results have shown that the FCS of left angular gyrus was lower but not reach significant in depression patients before ECT, comparing to the normal controls. It has also shown that FCS of left angular gyrus in depression patients increased significantly after the completion of ECT. The increased FCS of left angular gyrus may improve the ability of emotion regulation and lead to relief of depressive symptoms. And also, it should be noticed that the FCS of depression patients at 1 month after the completion of ECT decreased significantly comparing to the ending of ECT, without deterioration of depression. FCS is an index which reflects general functional connectivity between one brain region and the whole brain. For further studies, we need to explore the exact brain areas which contribute to the changes of FCS in left angular gyrus after ECT, and it may be conductive to clarify the relation between FCS in left angular gyrus and depression symptoms.

In this study, we also observed the change of cognitive function in depression patients. It has shown that general cognition and verbal fluency is impaired significantly after ECT, and recover one month after the completion of ECT. These results are consistent with previous study (Semkovska and McLoughlin, 2010). Many studies have shown that there is impaired cognition inclulding memory, verbal fluency and others, and it would be improved after successful psychotherapeutic, psychopharmacologic treatment. But if depression patients recive ECT, symptoms would be improved accompanied by reversible deterioration of cognition (Semkovska and McLoughlin, 2010). The reason is still unknow. Many studies have demonstrated left angular gyrus is involved in many cognitive tasks including executive function (Staresina and Davachi, 2006; Cabeza et al., 2008; Gauthier et al., 2009; Reineberg et al., 2015). The FCS of left angular gyrus in depression patients has increased after ECT, and it reversed in patients one month after ECT, which has shown the same tendency with general cognition, and verbal fluency. But no significant correlation between the changes in cognitive outcomes and FCS value of left angular gyrus were observed. So the relationship between the changed FCS of left angular gyrus and cognitive changes needs further studies to explore.

The limitations of this study also should be addressed. First, the patients recruited in the study took some medicines including anti-depressants and benzodiazepine. It should be noticed that these medicines may impact the fMRI results. Second, only bifrontal ECT was used in our study, and our results should be validated in other ECT protocols, such as bilateral ECT, right unilateral ECT and focal electrically administered seizure therapy.

In summary, current study have found that ECT significantly increase the FCS of left angular gyrus in depression patients, and the changed FCS in depression patients recovered obviously at 1 month after the completion of ECT. These results suggest that ECT could change functional connectivity of left angular gyrus in depression patients.

## Author Contributions

WX, ZX, and DZ recruited the patients. GJ, LW, and PH recruited the normal controls. ECT was performed by YC. XH and YY scanned the participants. QW, TB, and YC completed the clinical evaluation and cognitive tests, analyzed the data, and wrote the manuscript. YT and KW designed the study.

## Conflict of Interest Statement

The authors declare that the research was conducted in the absence of any commercial or financial relationships that could be construed as a potential conflict of interest.

## Footnotes

1 http://www.restfmri.net

2 www.fil.ion.ucl.ac.uk/spm
